# CH(II), a cerebroprotein hydrolysate, exhibits potential neuro-protective effect on Alzheimer’s disease

**DOI:** 10.1371/journal.pone.0222757

**Published:** 2019-09-23

**Authors:** Zehui Liu, Wanyan Wang, Tingyu Huang, Cunfang Wang, Ying Huang, Yong Tang, Jin Huang

**Affiliations:** 1 State Key Laboratory of Bioreactor Engineering, Shanghai Key Laboratory of New Drug Design, School of Pharmacy, East China University of Science and Technology, Shanghai, China; 2 Guangdong Long Fu Pharmaceutical Co., Ltd, Guangdong, China; 3 Guangdong Institute for Drug Control, Guangdong, China; 4 Department of Urology, Wuming Hospital of Guangxi Medical University, Guangxi, China; Texas Technical University Health Sciences Center, UNITED STATES

## Abstract

Alzheimer’s disease (AD) is a progressive neurodegenerative disorder, and is the most common type of cognitive impairment and dementia. There is a pressing need to improve the clinical efficacy and quality of life for AD patients, as limited treatments options for AD patients have been developed until now. In this study, we aim to investigate the protective effect of CH(II), a cerebroprotein hydrolysate consisted of abundant biological peptides, on preclinical model of AD. We found that CH(II) treatment effectively protects oxygen glucose deprivation (OGD)-induced N2A cell viability impairment and cell apoptosis. In addition, CH(II) significantly reduces H_2_O_2_-induced ROS accumulation and exhibits the protective activities against H_2_O_2_-induced oxidative injury. Intriguingly, we found that CH(II) treatment can effectively promote neurite outgrowth of N2A cells. Moreover, CH(II) obviously improve the cognitive and memorial function in scopolamine-induced amnesia mice model. Taken together, this study provides evidences of the neuroprotective activities of CH(II) and offers a potential therapeutic strategy for AD patients.

## Introduction

Alzheimer’s disease (AD) is a chronic neurodegenerative disorder which has become the most remarkable form of dementia among aging population [1]. It is characterized by memory and cognitive loss, as well as changes in personality and behavior [2]. It is reported that there are 50 million people living with dementia worldwide in 2018 and the number will increase triply to 152 million by 2050 [3], burdening healthcare systems and national economies. Since 1993, hundreds of attempts were made to develop effective drugs to cure AD but only five have been approved [4]. These approvals are consisted of four cholinesterase inhibitors (donepezil, tacrine, rivastigmine, galantamine), inhibiting messages sending between nerve cells and memorial keeping, and an N-methyl-D-aspartate (NMDA) receptor antagonist (memantine), preventing the damage to brain cells from excess glutamate [5–9],which provide limited treatment because of the simplex pharmacologic target and is far away to meeting the market demand. Therefore, considerable research for understanding the pathogenesis and exploring effective drugs of AD is extremely urgent.

Though the specific pathogenesis of AD has not been fully uncovered, there are various of AD pathophysiology been proposed including deposition of amyloid beta protein, abnormalities of cholinergic, oxidative stress and inflammatory [10–12], which may amplify and interact with each other in cycle of toxicity and lead to cell dysfunction, cell death and neuronal function [13]. Specifically, it is reported that the oxidative stress may be the earliest feature of AD, indicating the important role of free radicals in the progression of AD [14, 15]. Moreover, AD is characterized directly by brain atrophy owing to neuronal and synaptic loss, which cannot be repaired naturally [16]. Several studies aim to enhance cognitive and memorial level by increasing neurogenesis have proved the important role of neurogenesis in memorial improvements [17–20]. Consequently, the use of antioxidant and neurogenesis could be an alternative treatment for AD patients clinically.

Cerebroprotein hydrolysate is a mixture of peptides and free amino acids extracted from porcine brain tissue which has been proved to be effective in inhibiting microglial activation, neuro-inflammation and free radical formation and it has been shown to promote neuronal sprouting and stimulate neurogenesis [21–25]. Moreover, it can penetrate biological membranes easily and pass through the blood brain barrier to improve neuronal survivals, regulate neuronal plasticity and repair neurons [21, 26, 27]. Hence, cerebroprotein hydrolysate is widely regarded as a potential neurotrophic and neuroprotective drug in treatment of vascular dementia, traumatic brain injuries, ischaemic and AD in clinical [27–29].

In this study, we focus on a new cerebroprotein hydrolysate (II) (CH(II)), which is applied to the treatment of craniocerebral trauma and cerebrovascular diseases, brain trauma, post-cranial surgery, cerebrovascular disease sequelae associated with memory loss and attention deficit disorder, brain dysfunction, brain insufficiency in clinical in China. We aim to explore the potential application of CH(II) in the treatment of AD. We evaluated the neuro-protective effect of CH(II) on N2A cells and found that CH(II) treatment significantly protects cells against oxygen glucose deprivation (OGD)-induced cell death. In addition, CH(II) effectively impairs H_2_O_2_-induced ROS accumulation and shows the protective activities against H_2_O_2_-induced oxidative injury. Importantly, CH(II) obviously induces neurite outgrowth, suggesting the potential neurogenesis function of CH(II). Moreover, CH(II) could effectively improve cognitive and memorial function in scopolamine-induced amnesia mice model. Taken together, our study reveals the neuroprotective effects of CH(II) and provide a potential therapeutic approach in treating AD.

## Methods

### Cell culture

Neuro-2A (N2A, mouse neuroblastoma) cells were purchased from the Chinese Academy of Science (Shanghai, China) and maintained in DMEM (Gibco, NY, USA) supplemented with 10% (v/v) FBS (Gibco, NY, USA) and 1% penicillin/streptomycin in a humidified atmosphere containing 5% CO_2_ at 37°C. Cells were sub-cultured by trypsinization every 3 days when growing up to 75% confluence.

### Cell viability

Cells (5000 cells per well) were seeded into 96-well plates for 20 h, and then treated with different concentration of CH(II) from 0.001 to 1 μg/ml. CH(II) (H20051230) was kindly provided by Long Fu Pharmaceutical Co, Guangdong, China. After incubation of 48 h, cells were subjected to OGD or H_2_O_2_ for 20 h. Cells without OGD or H_2_O_2_ treatment were served as normal control and BDNF (for OGD) or NAC (for H_2_O_2_) were used as a positive control. Cell viability was measured by SRB assay as previously described[30]. Briefly, cells were fixed with 5%(w/v) trichloroacetic acid (TCA) for 1 h at 4°C. The excess dye was removed by washing with 1%(v/v) acetic acid for 5 times. The protein-bound dye was dissolved in 10 mM Tris buffer with 100 μl per well and the optical density was measured at 495 nm using microplate reader Synergy 2 (BioTek, VT, USA). Data are shown as mean ± SEM in three independent experiments.

### Neurite growth assay

N2A cells were plated into 6-well plates with 10^5^ cells per well and cultured in DMEM containing 10% FBS for 20 h. Then the medium was replaced by DMEM containing 10% FBS supplemented with CH(II) at a final concentration of 1 μg/ml for another 36 h to induce neurite outgrowth. The morphology of cells was observed under microscope (Olympus, Tokyo, Japan). Each well was calculated using four different fields. The average of neurite length of cells was measured by Image J as described previously [31]. Data are shown as mean ± SEM in three independent experiments.

### Cell apoptosis assay

Double staining for propidium iodide (PI) and Annexin V/FITC was performed to evaluate apoptosis in N2A cells as previously reported [32]. Cells were seeded into 6 wells plate with 10^5^ cells per well for 20 h and subjected to OGD/ H_2_O_2_ treatment with or without 1 μg/ml CH(II) for 24 h. cells were collected through trypsinization and washed with pre-cold phosphate-buffered saline (PBS) twice. Then cells were re-suspended in 350 μl 1x binding buffer (PBS supplemented with 1 mM EDTA and 2% FBS, pH 7.4) and incubated with 5 μl Annexin V-FITC in dark for 15 min followed by incubation of 5 μl PI in dark for 15 min at room temperature according to the manufacturer's instructions (AnnexinV-FITC Apoptosis Detection kit, eBioscience, MA, USA). The samples were analyzed by BD FACS Calibur flow cytometry (BD Biosciences, NJ, USA) with FlowJo software (BD Biosciences, NJ, USA). Annexin V/FITC-positive, PI-negative and Annexin V/FITC-negative, PI-positive were regarded to be in early and late apoptosis, respectively. The proportion of these two quadrant were calculated and shown as mean ± SEM.

### Western blotting

N2A cells were seeded into 6-well plates with 10^5^ cells per well for 20 h and then treated with OGD/ H_2_O_2_ with or without 1 μg/ml CH(II) for another 24 h. Cells were washed with PBS twice and lysed with RIPA buffer. Total cell lysates were collected and centrifuged at 12000 rpm for 15 min at 4°C, and supernatants were collected. The total protein concentrations were determined using BCA assay as described previously [33]. Samples were boiled in 100°C for 5 min, loaded on sodium dodecyl sulfate-polyvinylidene gel electrophoresis (SDS-PAGE) with 100 μg per well under constant voltage (90 V) and then transferred to the polyvinylidene difluoride (PVDF) membrane at 100 V, 300 mA for 2 h on ice. The membranes were blocked in 3% BSA-TBST buffer for 1 h at room temperature and probed with primary antibodies anti-caspase 3 (1:1000) at 4°C overnight. The blots were washed with TBST three times and incubated with secondary antibody (1:3000) for 3 h in room temperature. Afterwards, the blots were washed with TBST for 4 times and the immunoblots were detected using Pierce^™^ ECL Western Blotting Substrate. The relative density of cleaved caspase-3 and Map2 to GAPDH was determined using Image J and shown as mean ± SEM.

### Intracellular ROS accumulation Analysis

The production of ROS in cells was measured using Reactive Oxygen Species Assay Kit (Sigma-Aldrich, MO, USA) by confocal and flow cytometry [34]. N2A cells were seeded into 6-well plates with 10^5^ cells per well for 20 h and then treated with 1 μg/ml CH(II). After incubation for 24 h, cells were exposed to H_2_O_2_ (200 μM) for 1.5 h. As for fluorescence imaging, cells were treated with 10 μM DCFH-DA in vehicle medium in the dark for 30 min at 37°C followed by washing thrice with PBS buffer. The fluorescence density was performed by fluorescence microscope (A1R, NIKON, Japan). For flow cytometry, cells were collected through trypsinization and washed with pre-cold PBS twice. After that, cells were incubated with 10 μM DCFH-DA in vehicle medium in the dark for 30 min at 37°C followed by washing with PBS buffer for three times. The samples were analyzed using BD FACS Calibur flow cytometry (BD Biosciences, NJ, USA) in three independent experiments and FlowJo software.

### Cognitive ability assay

All the experimental procedures and animal in this study were approved and followed with the protocol formulated by the Animal Care and Use Committee at East China University of Science and Technology. C57BL/6 wild-type female mice (6–8 weeks) (JSJ, Shanghai, China) were used in the study to verify the cognitive ability of CH(II) as described previously [35]. Mice were raised in a quiet room at constant temperature (25°C) with 12 h light/dark cycle. During the experiments, all the mice were provided standard mice food and water. The effects of CH(II) on memory and cognitive were evaluated in a scopolamine-induced amnesia mice model using a passageway water maze (80 cm x 50 cm x 20 cm). The mice were separated into four groups with 5 per cages randomly and CH(II) were administered (i.v.) with 0.2 g/kg and 2 g/kg followed by injection of scopolamine (i.p.). Mice were put into water maze facing towards the wall of starting location and were trained to in search of the hidden platform and the latency time was recorded daily. Each mouse trained twice daily for 6 days. On day 7, mice were given a probe trial session where the platform in the pool was removed and mice were allowed to swim in search of it for 60 s. Swimming time and times crossing the target platform was recorded. The data was shown as mean ± SEM. The mice were put back to their cages after the tests and sacrificed after 24 h under sodium pentobarbital anesthesia.

### Statistical analysis

All values were shown as mean ± SEM of three independent experiments and replicated for three times. Statistical differences between two groups were analyzed using Student’s test in GraphPad Prism 5.0 software (GraphPad software, CA, USA). *P<0.05 was considered statistically significant.

## Results and discussion

### CH(II) improves cells viability following OGD or H_2_O_2_ treatment

**Fig 1:**
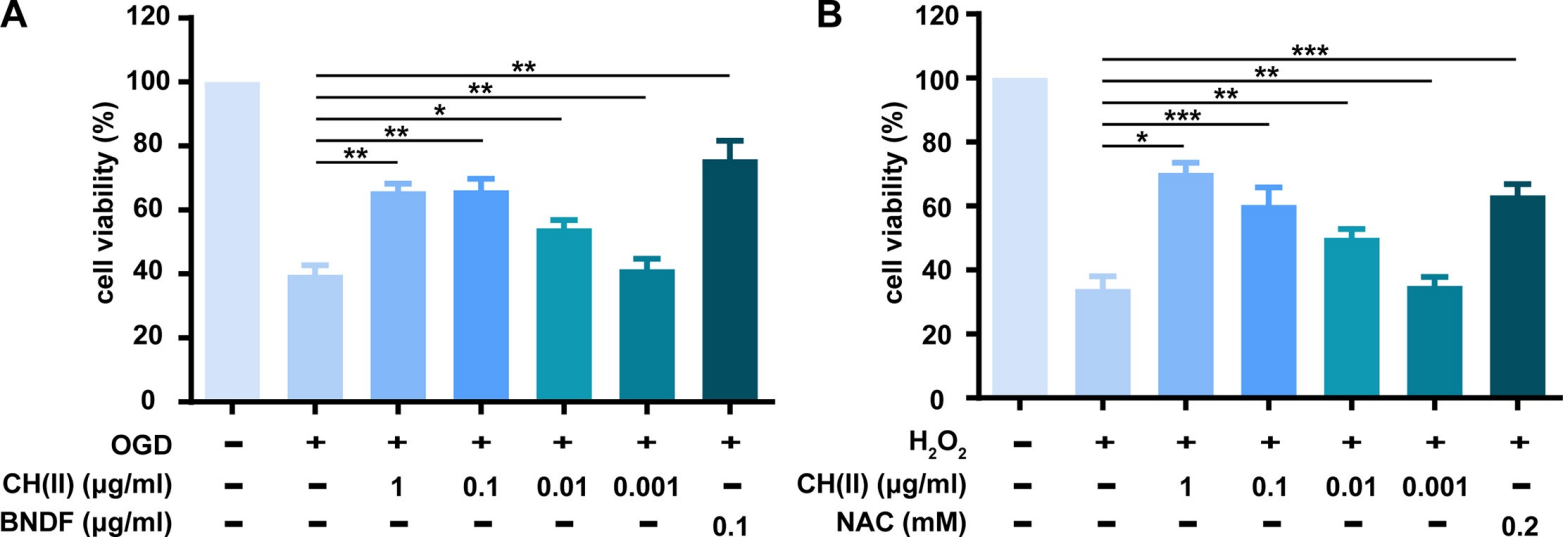
Effect of CH(II) on N2A cell viability. N2A cells were pretreated with different concentrations of CH(II) from 0.001 to 1 μg/ml or BNDF (0.1 μg/ml as positive for OGD) or NAC (0.2 mM as positive for H_2_O_2_) for 48 h followed treatment in OGD (A) or H_2_O_2_ (B) for another 20 h. The viability of cells was determined by the SRB assay. Data are shown as mean ± SEM in three independent experiments. Student’s t-test was displayed, *P<0.05, **P<0.01, ***P<0.001 versus OGD or H_2_O_2_. P<0.05 was considered statistical significantly.

Neuronal cells loss is the key pathological characteristics in AD. Oxygen-glucose deprivation (OGD) is an in vitro model to evaluate susceptibility ischemia caused by aging [36] and it preserves cell compositions including inflammatory competent cells, functional neurons, intercellular connections and locally released effectors [37], which consequently have been frequently used to model ischemic events and to study mechanism of neuro-protection and cell death [38]. Given that, we selected the OGD model to investigated the effects of CH(II) on N2A cells viability using SRB assay. Cells were treated with different concentration of CH(II) (0.001, 0.01, 0.1, and 1 μg/ml) and 0.1 μg/ml BNDF as positive for 48 h and then subjected to OGD for 24 h. As shown in Fig 1A, exposure to OGD caused a reduction on cell viability by about 60%. Under these conditions, pretreatment with CH(II) dose-dependent protected cells from OGD-induced cell death and increased cell viability to about 70% with 0.1 μg/ml. In addition, CH(II) could also improve cell viability exposed to H_2_O_2_ (Fig 1B), which indicates that CH(II) have protective effects on OGD-induced neuronal cell death.

### CH(II) protects cells against OGD-induced apoptosis

**Fig 2:**
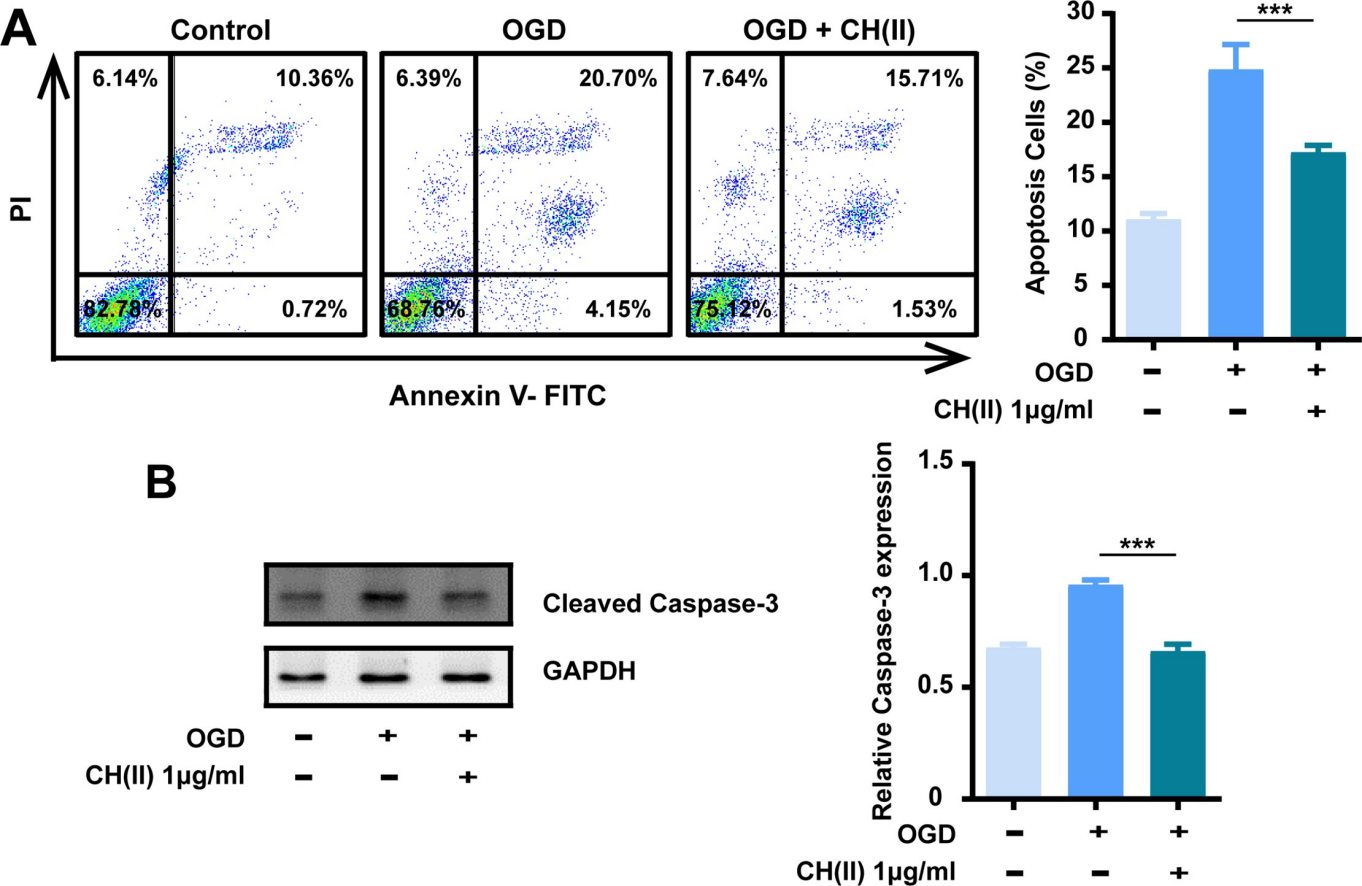
Effects of CH(II) on OGD-induced apoptosis in N2A cells. Cells were subjected to OGD treatment for 24 h with or without CH(II) (1 μg/ml). A: Anti-apoptosis of CH(II) was measured by flow cytometry. The proportion (%) of cells number is shown in each quadrant in control, OGD with or without CH(II) (1 μg/ml). B: Western blot analysis of apoptosis marker cleaved caspase-3 in N2A cells in control, OGD in the presence or absence of CH(II) (1 μg/ml) is shown. GAPDH was used as internal loading control. Relative density of cleaved caspase-3 to GAPDH was determined using Image J. Data are shown in three independent experiments. Student’s t-test was displayed, ***P<0.001 versus OGD. P<0.05 was considered statistical significantly.

Cell apoptosis is one of the reasons leading to neuronal cells dysfunction. We then turn to test the neuro-protective role of CH(II) on N2A cells using Annexin V-FITC/propidium iodide (PI) double staining assay. As shown in Fig 2A, OGD treatment promoted early and late apoptosis of N2A cells significantly while the pre-incubation of CH(II) remitted the OGD-induced cell damage and protected the cells, suggesting the anti-apoptosis activity of CH(II). Caspase-3 plays an important role in cell apoptosis and regarded as a biomarker of cell apoptosis [39]. We next examined the expression level of cleaved caspase-3 using western blot assay. As shown in Fig 2B, the density of cleaved caspase-3 compared to GAPDH increase obviously when exposed to OGD and pre-treatment of CH(II) suppressed the increase almost comparable with the control. These results indicated that CH(II) could protect cells against OGD-induced apoptosis.

### CH(II) protects cells against H_2_O_2_-induced apoptosis

**Fig 3:**
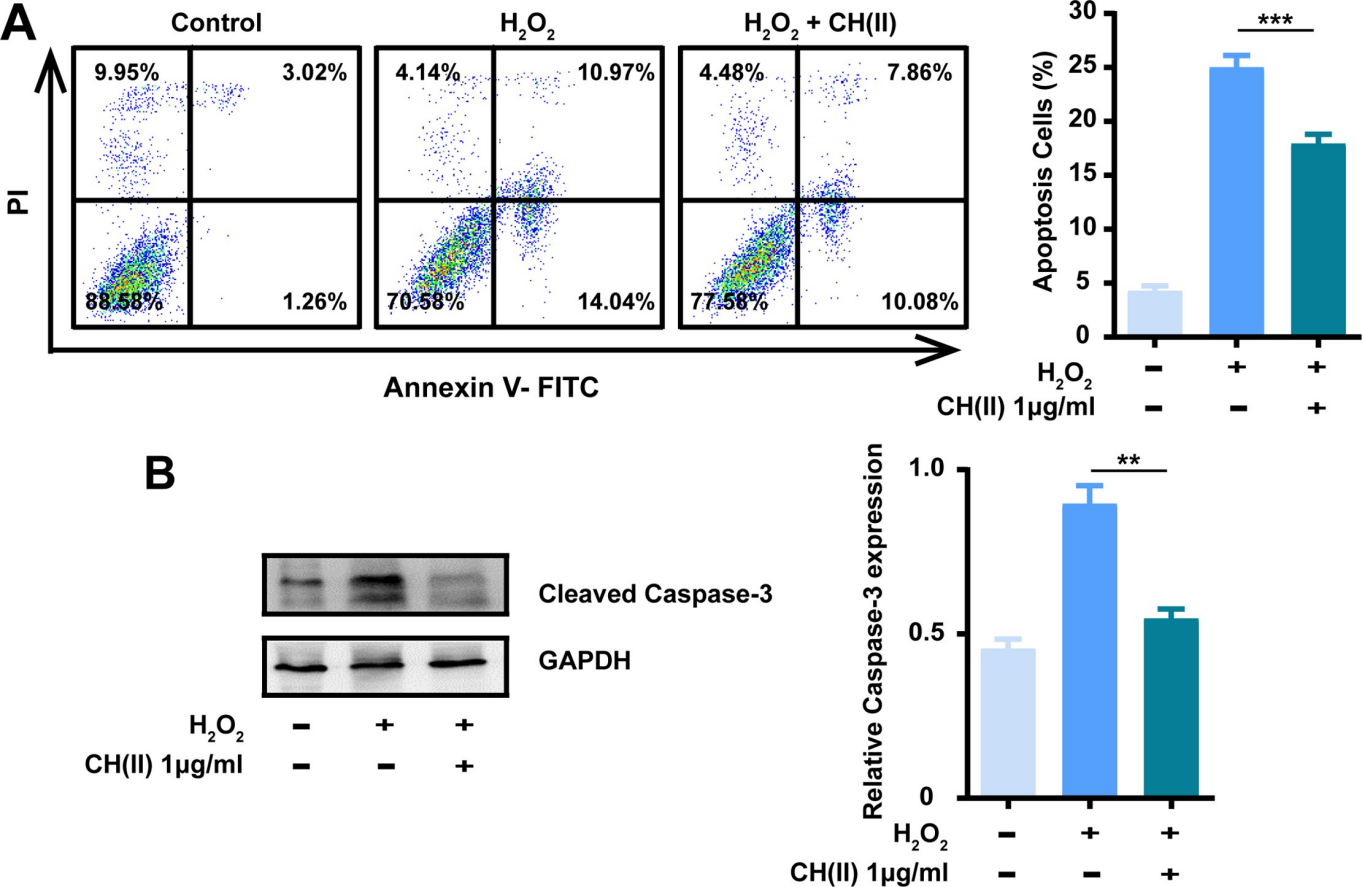
Effects of CH(II) on H_2_O_2_-induced apoptosis in N2A cells. Cells were treated with H_2_O_2_ for 24 h with or without CH(II) (1 μg/ml). A: Anti-apoptosis activity of CH(II) was measured by flow cytometry. The proportions (%) of cell number are shown in each quadrant in control, H_2_O_2_ with or without CH(II). B: Western blot analysis of apoptosis marker cleaved caspase-3 in N2A cells in control, H_2_O_2_ in the presence or absence of CH(II) (1 μg/ml) is shown. GAPDH was used as internal loading control. Relative density of caspase-3 to GAPDH was determined using Image J. Data were shown in three independent experiments. Student’s t-test was displayed, **P<0.01, ***P<0.001 versus H_2_O_2_. P<0.05 was considered statistical significantly.

Hydrogen peroxide (H_2_O_2_), a highly reactive oxygen species (ROS), gives rise to wide spread oxidative damage, and has been widely used to mimic oxidative stress and activate cell apoptosis in vitro [40, 41]. To further verify the anti-apoptosis effects of CH(II), apoptosis in N2A cells exposed to H_2_O_2_ was detected by flow cytometry and western blot. As expected, the number of apoptotic cells increased 20% after H_2_O_2_ treatment and the incubation of CH(II) remit the increasing by one-third, suggesting the function of CH(II) in preventing cell apoptosis (Fig 3A). Western blot assay indicated that the density of apoptosis marker cleaved caspase-3 increased double after H_2_O_2_ treatment (Fig 3B) but recovered almost equally to control while treated with CH(II). These results further confirmed that CH(II) have the ideal anti-apoptosis function and have the potential ability to prevent and treatment of AD.

### CH(II) prevents ROS accumulation in H_2_O_2_-induced N2A cells

**Fig 4:**
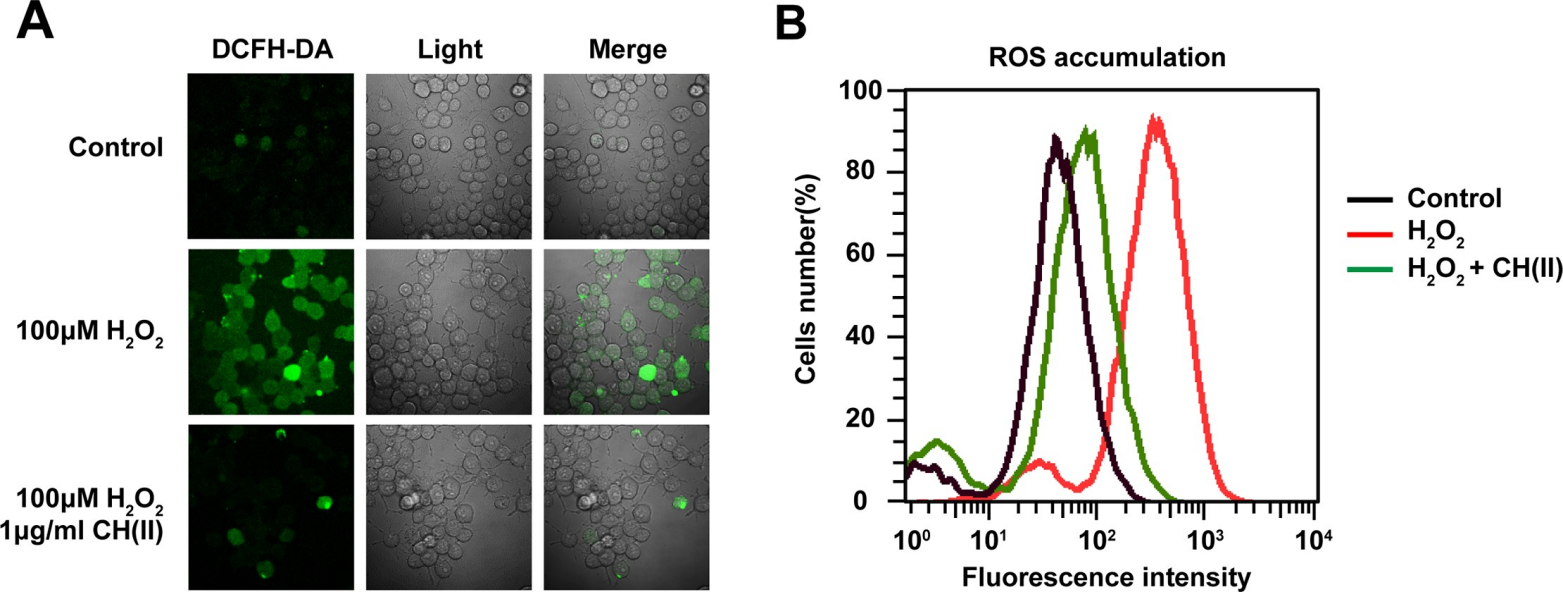
Effects of CH(II) on H_2_O_2_-induced ROS in N2A cells. Cells were treated with H_2_O_2_ for 24 h with or without CH(II) (1 μg/ml). A: Confocal was conducted against ROS marker, DCFH-DA, on nigral section (left) and light section (middle), and these two immunofluorographs were merged (right). B: The level of ROS was measured by flow cytometry in control (black) and H_2_O_2_ in the presence (green) or absence (red) of CH(II). The graph shows the DCFH-DA fluorescence intensity in three independent experiments.

Although the specific pathology of AD is far being understood, ROS generation is considered to be the most common origin in the progression of AD [42]. To explore whether CH(II) affect the H_2_O_2_-induced ROS generation in N2A cells, we used fluorescence imaging and fluorescence-activated cell-sorting (FACS) analysis to detect ROS levels. As for fluorescence, green fluorescence level from DCF under the microscopic images of N2A cells exposed to H_2_O_2_ with or without CH(II) exhibits the intracellular ROS generation. As shown in Fig 4A, the ROS level increased significantly after treatment with H_2_O_2_ for 1.5 h compared to control, while the pre-incubation of CH(II) in concentration of 1 μg/ml shown weak fluorescence intensity indicating the anti-oxidative activity of CH(II). In addition, FACS analysis indicated that the increased average fluorescence intensity of intracellular ROS accumulation induced by H_2_O_2_ was prevented by pre-incubation of CH(II) (Fig 4B). In summary, the results suggested that under H_2_O_2_-inuced oxidative stress, we observed significantly higher concentration of ROS in N2A cells and pre-treatment of CH(II) remit the oxidative stress in comparison to control, revealing possibility anti-oxidative mechanism in the neuronal protection effect of CH(II).

### CH(II) stimulates neurogenesis

**Fig 5:**
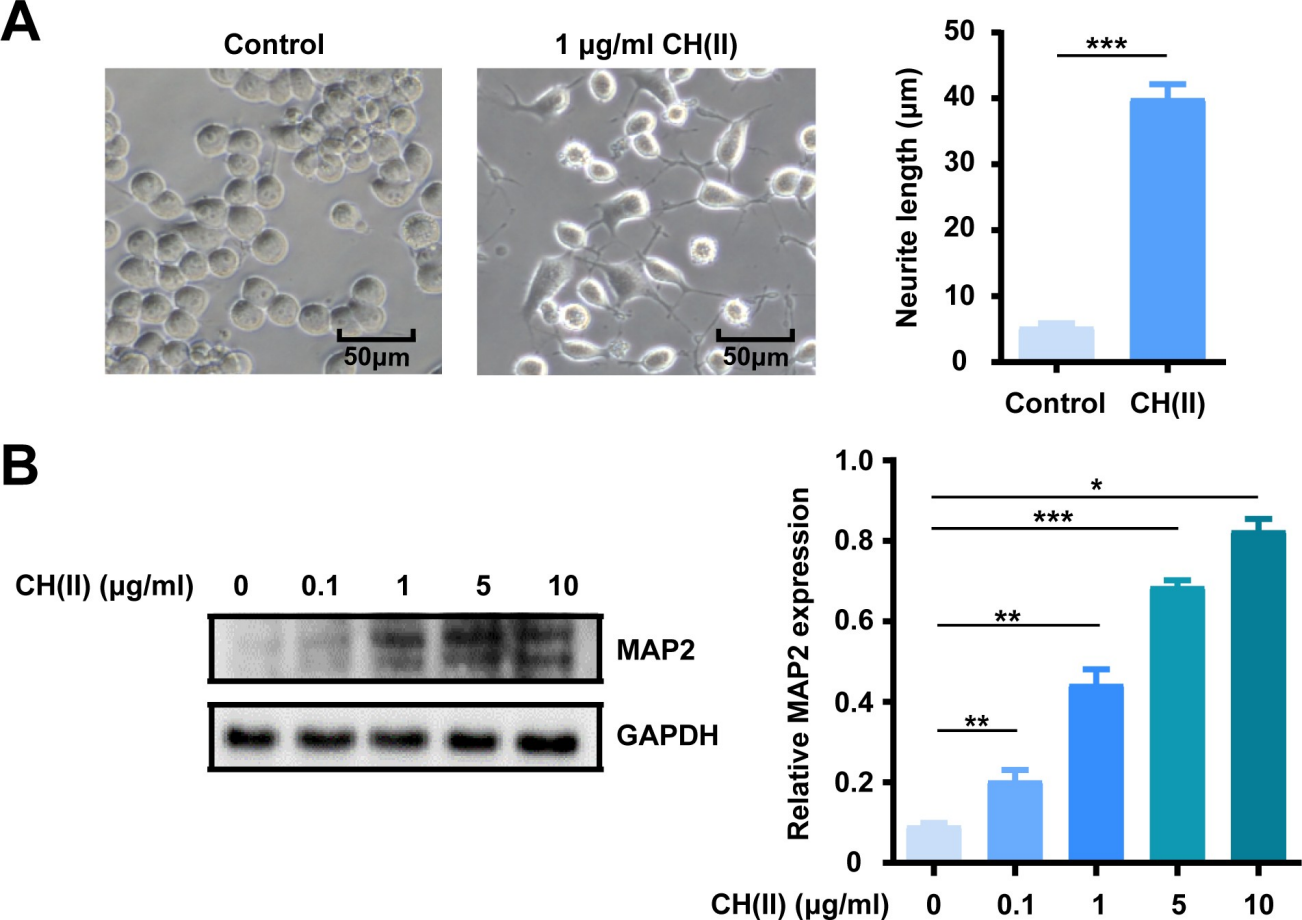
Effect of CH(II) on neurogenesis. A: Neurite growth assay of N2A cells with or without CH(II) (1 μg/ml) for 24 h was performed under a light microscope (magnification: x 200, bar: 50 μm). CH(II) significantly induced neuronal maturation of N2A cells by 80% compared with the vehicle treated control. The graph shows the neurite length of N2A cells as mean ± SEM, ***P<0.001. B: The level of neuronal marker Map2 in N2A cells treated with CH(II) in different concentrations for 24h was measured by western blot. GAPDH was used as internal loading control. Relative density of Map2 to GAPDH was determined using Image J. Data are shown in three independent experiments. Student’s t-test was displayed, **P<0.01, ***P<0.001 versus control. P<0.05 was considered statistical significantly.

Neuronal loss is the direct reason to the progression of AD and neurogenesis have been proved to play an important role in increasing cognitive and memorial level. Neuronal polarization such as neurites into dendrites is an essential step in the process of neuronal development and maturation and it is crucial for the correct transmit of electrical signals between neurons communication[31, 43]. However, there is non-pharmacologic therapies available for AD to stop or slow neurons damage and destruction, the origin factor making the disease fatal [44]. Given that, we tried to explore the neurogenesis activity of the CH(II) by observing the neurite formation of N2A cells in response to the treatment of the CH(II). As shown in Fig 5A, treatment of CH(II) for 36 h significantly stimulated the neuronal polarization of N2A cells and the average neurite length increased about 80% compared to control. Moreover, the terminal of most neurites exhibited a backwards movement observed by the short minor neurite length. MAP2 (microtubule-associated protein 2), a widely used neuronal dendritic marker, was strongly upregulated in the presence of CH(II) (Fig 5B). These results indicate that CH(II) has a positive effect on neurite outgrowth relieving irreversible neuros damage and destruction in the brain, which suggested the potential neurogenesis function of CH(II) in the treatment of AD.

### CH(II) improves cognitive function in a mice model of AD

**Fig 6:**
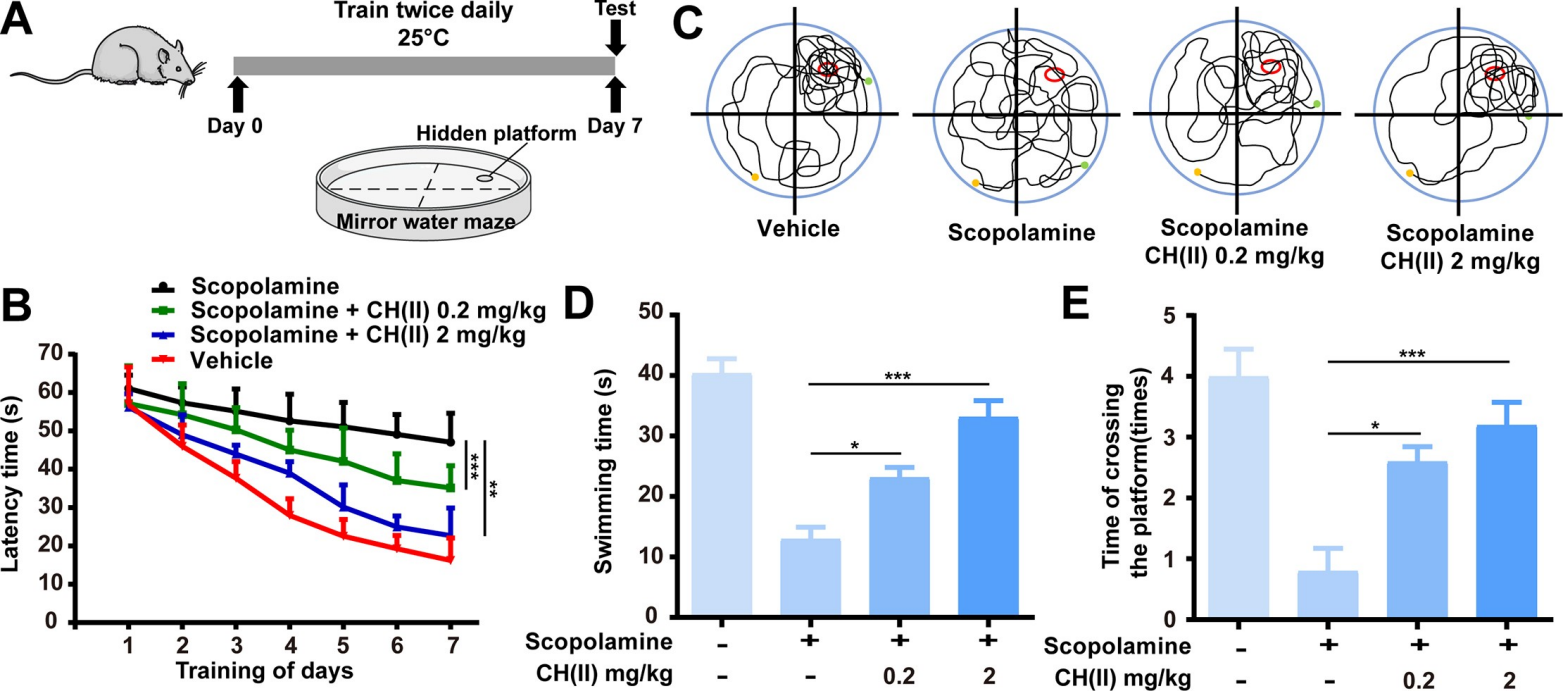
Effects of CH(II) on behavioristics in AD mice model. Effects of CH(II) on scopolamine-induced memorial deficit was measured using water maze tests. CH(II) was administered through tail vein injection followed by injection of scopolamine daily and trained for 6 days. A: Experimental schedule to evaluate the effects of CH(II) on behavioristics. B: Mean escape latency to the hidden platform for the four groups over 7 days in the water maze test. C: Representative path tracing during the probe trials on day 7. D, E: Time spent in the targeted quadrant (D), and the times crossing the targeted platform (E) on day 7 during the cognitive ability test. All the Values are expressed as mean ± SEM. *P<0.05, **P<0.01, ***P<0.001 versus scopolamine group. P<0.05 was considered statistical significantly.

The effect of CH(II) on cognitive improvements was assessed in scopolamine-induced AD animal model using water maze. Scopolamine can antagonize the muscarinic receptor and block the cholinergic pathway [45], which is widely used as a typical AD model to explore the potential drug candidates [46]. All the mice were separated into 4 groups with 5 per cages randomly and trained twice daily for 6 days and received cognitive ability test in water maze on the seventh day. As shown in Fig 6B, the scopolamine group exhibited longer swimming time than the control group during the training days. CH(II) administration to mice through tail vein injection at a dose of 0.2 or 2 mg/kg as described significantly reduced average swimming times prolonged by scopolamine. In addition, CH(II) prolonged the swimming time in the target quadrant and increased the times crossing the target platform in cognitive ability test compared to control with concentration-dependent manner (Fig 6C–6E). Although the effect of CH(II) on other AD mice model is need to be further investigated, the effectivity of CH(II) in scopolamine-induced AD mice suggests it is potential anti-AD therapy.

## Conclusions

In summary, we have demonstrated that the neuroprotection and neurotrophication effect of CH(II) in neuronal cell model and scopolamine-induced amnesia mice model. Specifically, CH(II) treatment effectively prevent the N2A cells from OGD- and ROS- induced cell death and significantly promotes neurite outgrowth in N2A cells. Importantly, CH(II) administration obviously improve cognitive and memorial function in scopolamine-induced mice model. These compelling data suggest that CH(II) is a potential therapeutic candidate for patients with AD and warrant an early phase trial to clarify an optimal dosage regimen for AD.
